# Supplementary figures and images for: Genetic Channelization Mechanism of Four Chalcone Isomerase Homologous Genes for Synergistic Resistance to Fusarium wilt in Gossypium barbadense L
Source: Int J Mol Sci. 2023 Sep 30;24(19):14775. doi: 10.3390/ijms241914775 (PMC10572676; doi:10.3390/ijms241914775)

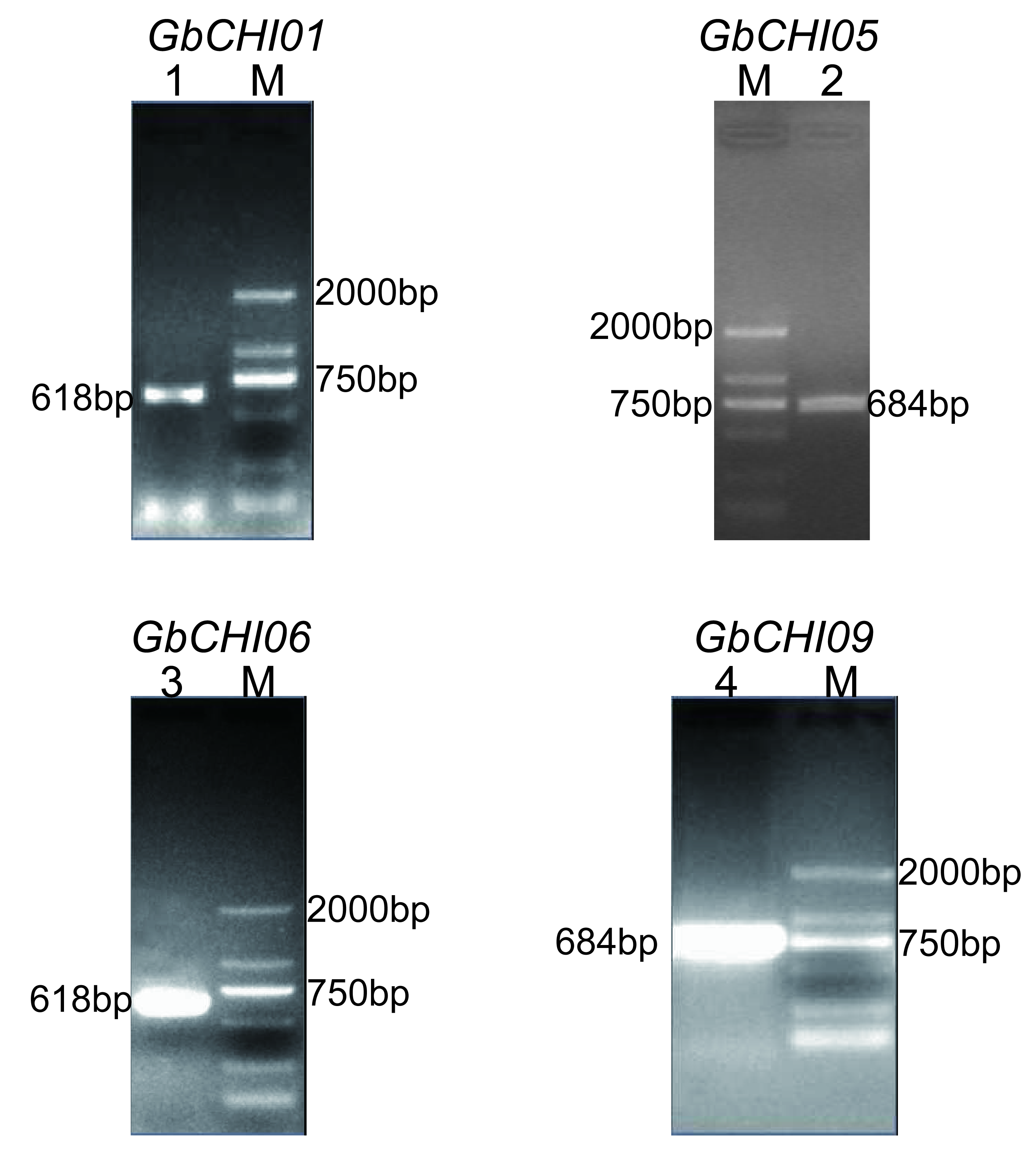

Supplement: Supplementary file 1 [file ijms-24-14775-s001.zip › supplementary files/Fig S1.tif]

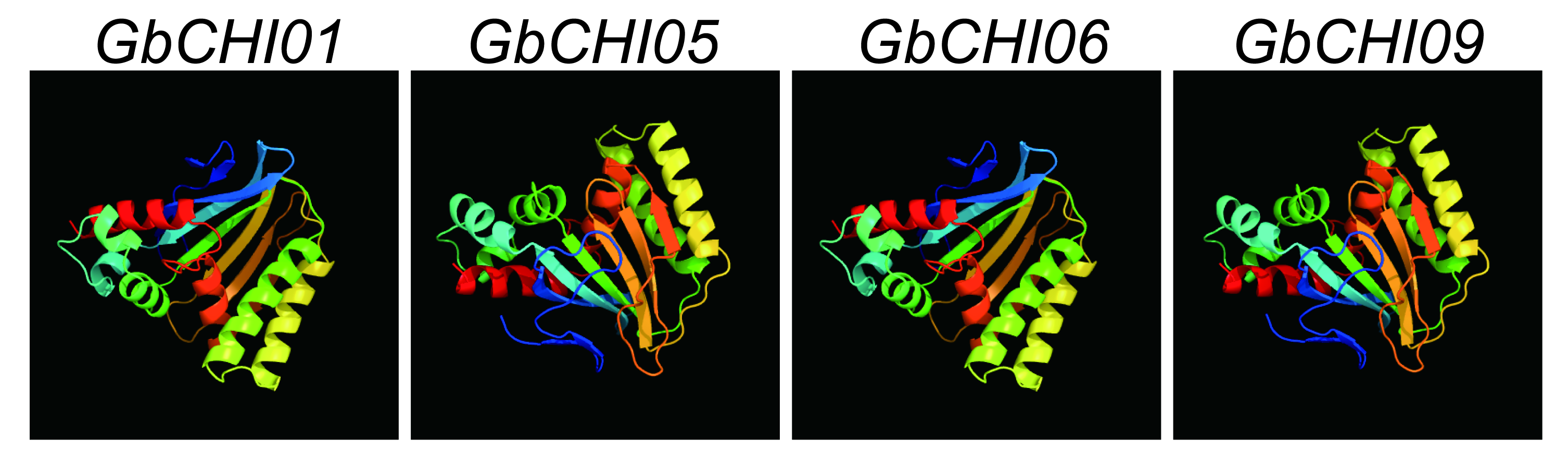

Supplement: Supplementary file 1 [file ijms-24-14775-s001.zip › supplementary files/Fig S2.tif]

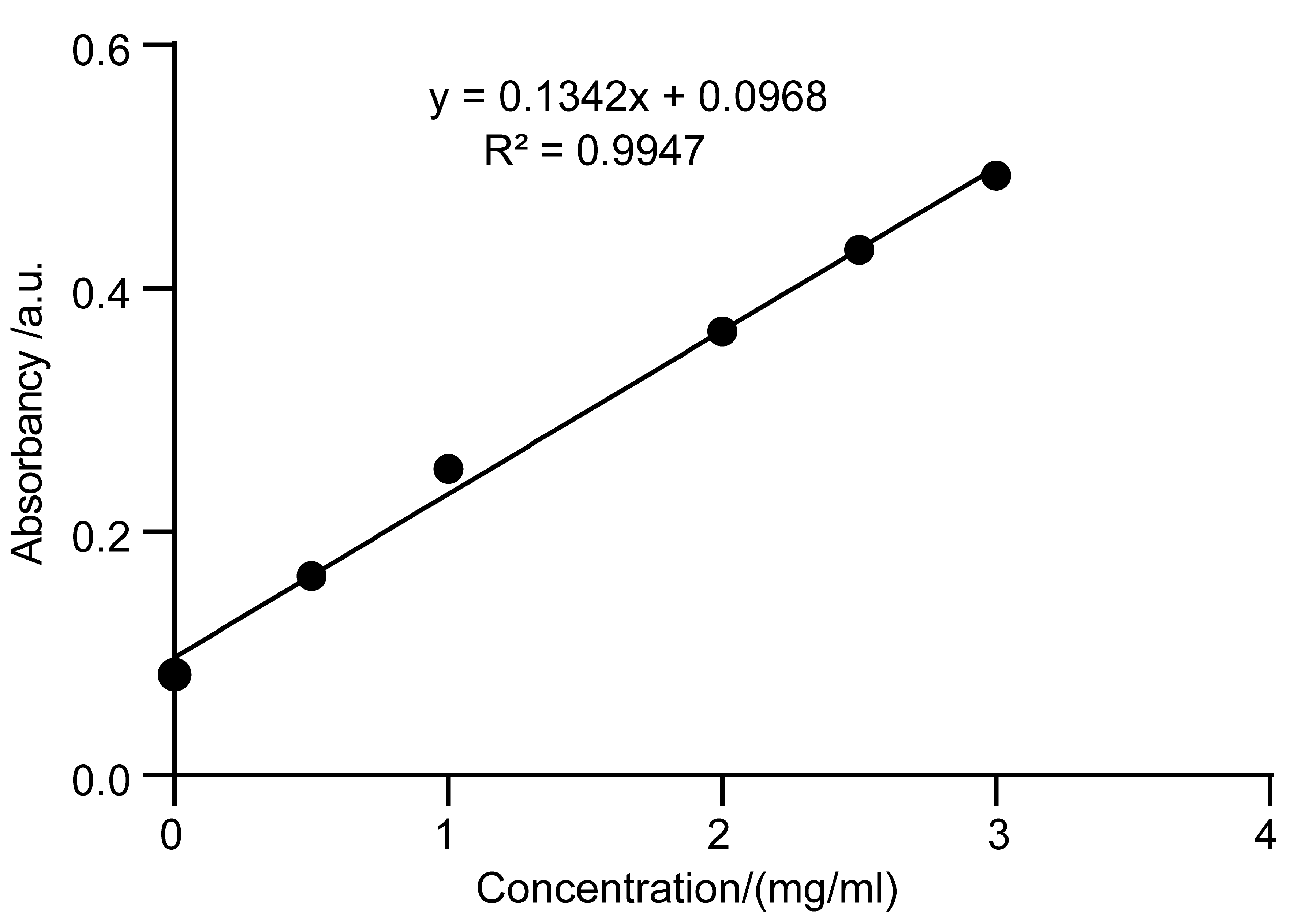

Supplement: Supplementary file 1 [file ijms-24-14775-s001.zip › supplementary files/Fig S4.tif]

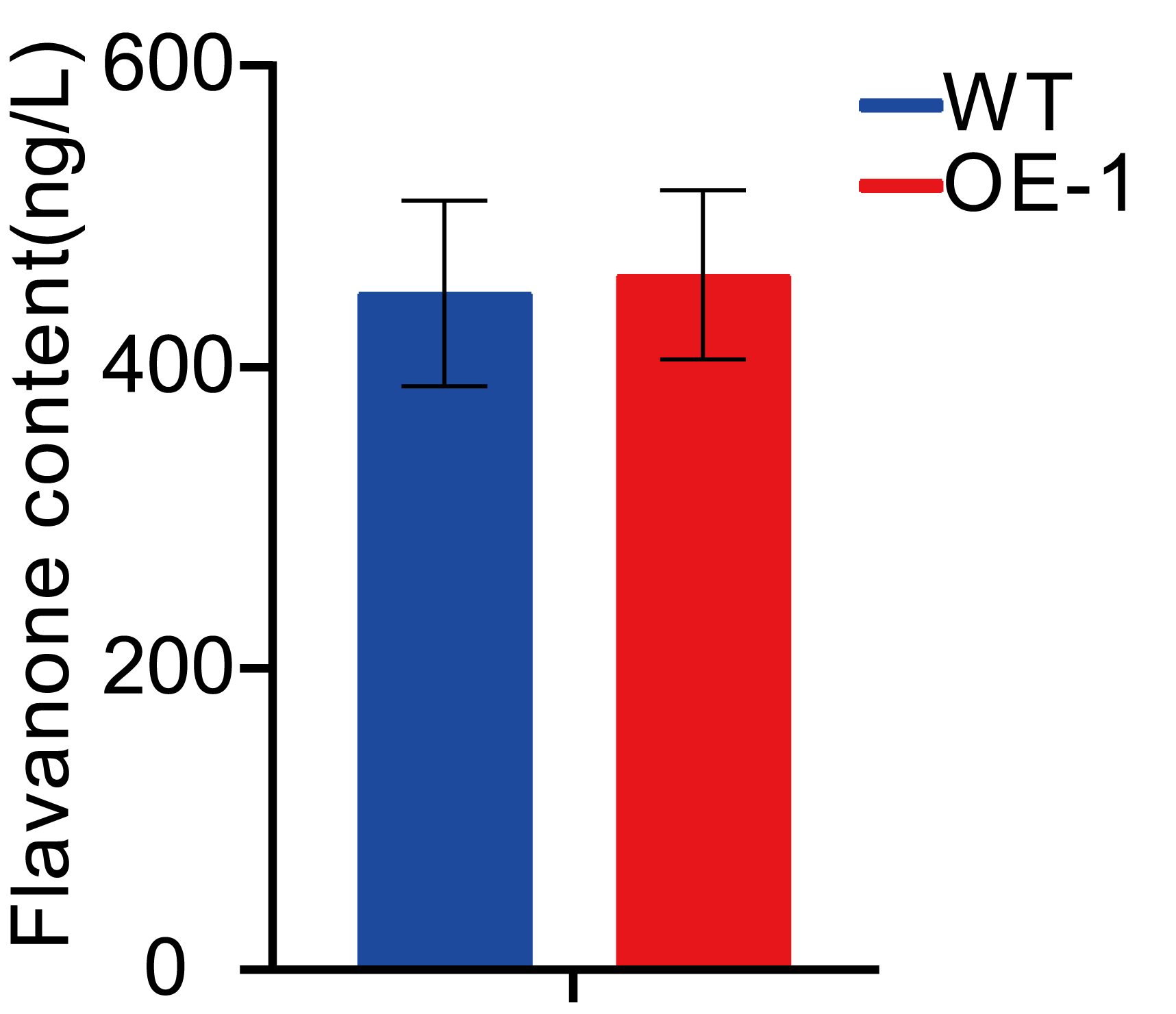

Supplement: Supplementary file 1 [file ijms-24-14775-s001.zip › supplementary files/Fig S5.tif]
